# Associations of urinary sodium excretion with central hemodynamics and changes in vascular structure and function at high altitude

**DOI:** 10.1111/jch.14356

**Published:** 2021-09-03

**Authors:** Zhipeng Zhang, Hang Liao, Xin Zhang, Qingtao Meng, Rufeng Shi, Jiayue Feng, Xinran Li, Qiling Gou, Runyu Ye, Xianjin Hu, Xiaoping Chen

**Affiliations:** ^1^ Department of Cardiology West China Hospital Sichuan University Chengdu China

**Keywords:** central hemodynamic, high altitude, sodium intake, vascular structure and function

## Abstract

Research reports on associations of urinary sodium excretion with central hemodynamic parameters and vascular changes are quite limited in general or non‐hypertensive population. The purpose of the current study was to explore such associations in Chinese general Tibetans living at high altitude. This cross‐sectional study was conducted in Luhuo County, Ganzi Tibetan Autonomous Prefecture with average elevation of 3800 meters from December 2018 to January 2019. A total of 294 Tibetans were included in the current study. Twenty‐four hour urinary sodium excretion was estimated by second fasting spot urine in the morning using Kawasaki formula. Central hemodynamic parameters, including central systolic blood pressure (CSBP), central diastolic blood pressure (CDBP), central pulse pressure (CPP), central mean arterial pressure (CMAP), augmentation pressure (AP), and augmentation index standardized for heart rate of 75 (AIx_75_), were evaluated using the SphygmoCor system. Vascular structures and functions were assessed by carotid intima media thickness (CIMT) test and brachial ankle pulse wave velocity (baPWV), respectively. Estimated mean 24h urinary sodium excretion of Tibetans in Luhuo County was 5.26±1.61 g. After adjustment, estimated 24h urinary sodium was positively associated with CSBP (β = 1.15, *p* = .008) and CPP (β = 0.87, *p* = .013). Line graph of means across urinary sodium quartiles showed that associations of 24 h urinary sodium excretion with AIx_75_ and baPWV presented approximate “J” shape after controlling for confounders. Estimated 24 h sodium excretion was independently and positively associated with CSBP and CPP. Moreover, association between urinary sodium excretion and arterial elasticity, as evaluated by baPWV and AIx_75_, presented “J” shape. Further studies are needed to verify J‐shaped association and “safe” zone of sodium intake.

## 1 INTRODUCTION

Previous studies reported that high‐salt diet is among the most important contributing factors to pathogenesis of hypertension and leads to vascular remodeling and arteriosclerosis through inflammatory responses, oxidative stress, and activation of renin‐angiotensin‐aldosterone system (RAAS).^1^ Remodeling of central and peripheral arteries increases amplitude of forward and reflected waves resulting in elevation of central blood pressure. Central arterial pressure represents direct pressure load exposed to left ventricle and has a substantial impact on perfusion of coronary artery and brain. Previous studies revealed that central arterial pressure is more closely correlated with vascular diseases, target organ damage of hypertension, and cardiovascular (CV) events compared to brachial artery pressure.^2^, ^3^

There has been little focus on associations of salt intake with central arterial pressure and vascular changes, especially in general population and individuals without hypertension. In addition, findings of existing studies are inconsistent depending on different populations, average amount of salt intake, and status of blood pressure. In particular, no study focusing on such association has been reported in people living at high altitude areas. Low oxygen availability at high altitude imposes extreme physiological challenges on aerobic organisms. Hypoxic environment leads to impairment of endothelial function (arising from altered sympathetic nerve activity, shear stress, oxidative stress, and inflammation^4^) which is closely related to hypertension and arterial stiffness. In this context, whether associations of salt intake with central blood pressure and vascular changes in hypoxic environment still exist or present different traits compared to residents living at lower altitude remain to be explored. This is important because dietary sodium plays roles in lifestyle modification in management of hypertension and CV diseases.

Luhuo County is located in north‐central part of Ganzi Tibetan Autonomous Prefecture, Sichuan province, China, with an average elevation of 3800 meters and climate of Tibetan Plateau. It is a multi‐ethnic area with total population of about 40,000 people, most of whom are Tibetans. The purpose of the current study was to explore associations of sodium intake with central hemodynamics and vascular changes among general Tibetans in Luhuo County.

## 2 METHODS

### 2.1 Study design and population

This cross‐sectional study was carried out in Luhuo County, Ganzi Tibetan Autonomous Prefecture, Sichuan province, China from December 2018 to January 2019. Researchers firstly numbered every township in consecutive Arabic numbers with help of local staff members of Centers for Disease Control. Four townships were then randomly selected for the current study using computer. A total of 383 native Tibetans, who gave their informed consent were recruited for study. A total of 294 participants were included in the current study after excluding participants with missing values and fulfilling exclusion criteria. The current study was approved by Ethics Committee of Sichuan University and complied with Helsinki Declaration.

Inclusion criteria included adults aged 18–80 years old. Exclusion criteria included: highly suspected secondary hypertension, hypertension emergency, severe arrhythmia including atrial fibrillation, severe peripheral artery disease, valvular heart diseases, previous history of severe CV, and cerebrovascular diseases, serum creatinine > 150 μmol/L, autoimmune disease, malignant tumors, and other circumstances that were unsuitable for participation in the study.

Investigations on demographics, history of cigarette smoking and alcohol consumption, lifestyle, diet, medical history, and medications in addition to physical examination were conducted by trained staff members with help of local health workers, who undertook language translation.

### 2.2 Measurement of urinary sodium

Second spot urine of patients was collected after about 12 h of fasting and centrifuged between 8:00 a.m. and 9:00 a.m. Upper layer of urine was stored in cryopreservation tubes and then transferred to West China Hospital in cool boxes. Concentrations of urinary sodium, potassium, chloride, and creatinine were determined using automatic analyzer (Hitachi DXC800) and 24 h urinary sodium was estimated using Kawasaki formula^5^ which is presented in **Supplementary material**.

### 2.3 BaPWV measurement

BaPWV is a noninvasive method for detecting arterial stiffness, which has been validated with carotid femoral pulse wave velocity (cfPWV)^6^ and invasive PWV.^7^ BaPWV has been used widely in prospective studies for prediction of CV diseases^8^ and all‐cause mortality,^9^ mainly in Asia.

BaPWV examination was conducted using Omron oscillometry‐based device (VP1000 BP‐203RPE‐III, ColinCo, Ltd, Komaki, Japan) in warm and quiet room. After 5‐min rest in supine position, four cuffs were wrapped in bilateral upper arms and ankles of patients, two ECG pads were clipped to both wrists and one heart sound sensor was placed at the corner of sternum. Pulse wave velocity was computed automatically based on estimated distances and time consumed between sampling points. Average values of left and right sides were used for analysis.

### 2.4 Carotid intima‐media thickness (CIMT) test

CIMT test was undertaken by trained ultrasound physician using Philips portable carotid ultrasonography system (Philips CX‐50). Study patients sat in supine positions with their heads deviating towards opposite side to detector during examination. Measurements were made in anterior and posterior wall of common carotid artery at approximate distance of 10 mm from bifurcation on both sides. Mean values of anterior and posterior wall of both sides were computed for analysis.

### 2.5 Central hemodynamics

Parameters of central hemodynamics, including CSBP, central diastolic blood pressure (CDBP), central pulse pressure (CPP), central mean arterial pressure (CMAP), augmentation pressure (AP), and AIx, were noninvasively determined by applying proprietary digital signal processing and transfer function using cuff‐based SphygmoCor system (AtCor Medical, Sydney, New South Wales, Australia).^10^, ^11^ After 5‐min rest on chairs with backrests, cuffs were wrapped on dominant upper arms of patients. Brachial blood pressure and wave form were automatically determined and transferred into aortic pressure wave form using generalized transfer function provided by SphygmoCor software.^11^ AIx_75_ was computed to standardize for heart rate of 75 beats/min.

### 2.6 Statistical analysis

Quantitative data were presented as mean ± SD or median (P_25_, P_75_) and qualitative data were presented as percentages or frequencies. Continuous variables in different groups were compared using analysis of variance. Differences in categorical variables among groups were compared using χ^2^ tests. Partial correlation analysis was used to analyze correlations of estimated 24 h sodium intake with central hemodynamic parameters, baPWV and CIMT after controlling for age, sex, smoking status, alcohol consumption, total cholesterol (TC), fasting blood glucose (FBG), waist circumference (WC), uric acid (UA), heart rate (HR), antihypertensive therapy, and lipid‐lowering therapy (AIx_75_ was additionally adjusted for height). Variables that presented significant correlation with estimated 24 h sodium intake were included in multiple linear regression model as dependent variables and estimated 24 h sodium intake was analyzed as an independent variable. Adjusted confounders in multiple linear regression model included significant variables in univariate linear regression (*p* < .1) and that known to be correlated with dependent variables. Line graph of means across urinary sodium excretion quartiles were drawn to visualize relationship between urinary sodium excretion with dependent variables before and after controlling for confounders. *p* < .05 was considered statistically significant.

## 3 RESULTS

### 3.1 Basic characteristics

A total of 383 patients were recruited and 294 of them were included in the current study (four patients with previous history of stroke, 22 patients with previous history of coronary artery diseases, one patient with serum creatinine level > 150 μmol/L and 62 patients with incomplete data). Mean age of patients was 42.3 ± 10.8 years, 32.3% were males (mean age: 46.9 ± 13.2 years) and 67.7% were females (mean age: 40.2 ± 8.7 years). SBP/DBP were 129.7 ± 18.1/81.9 ± 13.0 mm Hg, estimated 24 h urinary sodium excretion was 5.26 ± 1.61 g and estimated 24 h salt intake was 13.36 ± 4.09 g. Basic characteristics of study patients across urinary sodium excretion quartiles are presented in Table 1.

**TABLE 1:** Basic characteristics of the patients across the urinary sodium quartiles

	Total	Q1	Q2	Q3	Q4	*p* value
Age (years)	42.30±10.78	42.97±11.09	42.39±10.40	40.31±10.04	43.60±11.49	.296
Smoking [n (%)]	29 (9.9)	8 (10.8)	7 (9.6)	9 (12.3)	5 (6.8)	.709
Alcohol [n (%)]	32 (10.9)	9 (12.2)	5 (6.8)	11 (15.1)	7 (9.5)	.420
DM [n (%)]	14 (4.76)	6 (8.22)	2 (2.70)	4 (5.41)	2 (2.74)	.342
HTN [n (%)]	84 (28.57)	22 (30.14)	20 (27.03)	23 (31.08)	19 (26.03)	.889
BMI (kg/m^2^)	26.02±4.48	24.60±4.65	25.71±4.59	26.69±4.25	27.07±4.07	.003
WC (cm)	82.96±13.16	80.54±12.03	80.66±12.67	84.55±13.18	86.09±14.06	.018
OSBP (mm Hg)	129.7 ±18.1	126.1±17.8	129.9±18.8	128.6±17.7	134.2±17.4	.052
ODBP (mm Hg)	81.9±13.0	82.4±12.6	80.5±13.7	82.5±12.4	82.3±13.4	.748
CSBP (mm Hg)	118.5±15.9	114.9±15.1	120.0±16.9	116.0±15.1	123.5±15.5	.006
CDBP (mm Hg)	83.5±11.8	81.8±11.9	84.5±12.4	84.0±10.8	83.8±12.3	.554
HR	84.0±20.1	90.2±34.4	79.7±11.7	84.2±10.8	81.8±11.0	.011
AIx75	26.4±13.6	27.0±14.8	28.7±14.6	25.5±11.3	24.4±13.4	.276
CPP (mm Hg)	35.0±9.9	33.1±8.9	35.4±10.1	32.0±8.2	40.0±10.6	<.001
baPWV (cm/s)	1475.7±361.1	1547.7±485.2	1462.0±302.4	1435.8±276.9	1454.0±336.5	.248
IMT (mm)	0.63±0.14	0.65±0.14	0.63±0.15	0.59±0.12	0.66±0.15	.033
FBG (mmol/l)	4.45±1.80	4.57±2.09	4.26±1.08	4.40±1.60	4.55±2.20	.728
Crea (μmol/l)	63.8±15.9	67.2±17.2	62.0±15.2	63.5±14.1	62.5±16.9	.201
UA (μmol/l)	274.5±92.6	286.5±95.3	251.8±89.0	282.6±87.0	276.8±96.7	.115
TC (mmol/l)	5.09±1.13	5.09±0.96	5.17±1.06	4.78±1.17	5.32±1.27	.038
TG (mmol/l)	1.35±0.85	1.36±0.63	1.21±0.80	1.30±0.87	1.55±1.05	.087
HDL‐C (mmol/l)	1.54±0.39	1.50±0.38	1.56±0.30	1.60±0.56	1.52±0.27	.551
LDL‐C (mmol/l)	3.19±0.89	3.13±0.84	3.18±0.94	3.13±0.80	3.34±0.93	.461
UNa (mmol/l)	122.1±72.4	106.3±61.9	125.3±59.3	139.5±74.5	117.6±87.5	.042
UK (mmol/l)	44.4±28.1	64.7±31.0	47.8±24.2	39.0±19.8	26.1±21.1	<.001
24h UNa (g/day)	5.26±1.61	3.31±0.66	4.70±0.29	5.67±0.34	7.35±1.02	<.001
24h UK (g/day)	2.24±0.46	1.95±0.40	2.17±0.35	2.29±0.45	2.56±0.41	<.001
Antihypertensive therapy [n (%)]	17 (5.8%)	6 (8.2%)	6 (8.1%)	3 (4.1%)	2 (2.7%)	.365
Lipid‐lowering therapy [n (%)]	3 (1.0%)	1 (1.4%)	1 (1.4%)	0 (0%)	1 (1.4%)	.796

*Abbreviations*: DM, diabetes mellitus; HTN, hypertension; BMI, body mass index; WC, waist circumference; OSBP, office systolic blood pressure; ODBP, office diastolic blood pressure; HR, heart rate; TC, total cholesterol; TG, triglyceride; HDL‐C, high density lipoprotein; LDL‐C, low density lipoprotein; FBG, fasting blood glucose; Crea, creatinine; UA, uric acid; baPWV, brachial ankle pulse wave velocity; CSBP, central systolic blood pressure; CDBP, central diastolic blood pressure; CPP, central pulse pressure; AIx75, augmentation index standardized for heat rate 75; cIMT, carotid intima media thickness; UNa, urine sodium; UK, urine potassium; UCl, urine chloride; UCrea, urine creatinine; 24h UNa, 24h urine sodium; 24h UK, 24h urine potassium.

### 3.2 Associations of urinary sodium excretion with peripheral and central hemodynamics

Partial correlation analysis in the current study showed that estimated 24 h urinary sodium excretion was positively correlated with office SBP (r = 0.178, *p* = .005) and office PP (r = 0.195, *p* = .002) but it was not correlated with office DBP (r = 0.029, *p* = .651) and office MAP (r = 0.099, *p* = .122).

In addition, partial correlation analysis showed that estimated 24 h urinary sodium excretion was positively correlated with CSBP and CPP (r = 0.176, *p* = .006 and r = 0.176, *p* = .011, respectively). However, correlations of estimated 24h urinary sodium excretion with CDBP, CMAP, and Ln AP (*p* = .380, .069, and .465, respectively) were not significant. Estimated 24h urinary sodium excretion was significantly and negatively correlated with AIx_75_ (*p* = .036). These findings are presented in Table 2. Multiple linear regression analysis showed that 24 h urinary sodium excretion was independently and positively associated with CSBP and CPP (β = 1.15, *p* = .008 and β = 0.87, *p* = .013, respectively). However, associations of estimated 24 h urinary sodium excretion with CDBP, CMAP, and Ln AP were not significant. Findings of the current study also established that 24 h urinary sodium excretion was significantly and negatively associated with AI_X75_ (β = ‐1.12, *p* = .036) (Table 3).

**TABLE 2:** Partial correlation analysis between 24h urinary sodium with central hemodynamic parameters, baPWV, and cIMT

	Correlation coefficient	*p* value
CSBP	0.176	.006*
CDBP	0.057	.380
CPP	0.165	.011*
CMAP	0.118	.069
Ln AP	‐0.049	.465
AIx75	‐0.136	.036**
baPWV	‐0.178	.005*
cIMT	‐0.098	.168

*Abbreviations*: CSBP, central systolic blood pressure; CDBP, central diastolic blood pressure; CPP, central pulse pressure; CMAP, central mean arterial pressure; AIx75, augmentation index standardized for heat rate 75; cIMT, carotid intima media thickness; BaPWV, brachial ankle pulse wave velocity; TC, total cholesterol; FBG, fasting blood glucose; WC, waist circumference; UA, uric acid; HR, heart rate; MAP, mean arterial pressure.

*Statistically significant after adjusting for sex, age, smoking status, alcohol consumption, TC, FBG, WC, UA, HR, peripheral MAP, antihypertensive therapy, and lipid‐lowering therapy.

**Statistically significant after adjusting for sex, age, smoking status, alcohol consumption, TC, FBG, WC, height, UA, antihypertensive therapy, lipid‐lowering therapy, and peripheral MAP.

**TABLE 3:** Multiple linear regression analysis between 24h urinary sodium with central hemodynamic parameters, baPWV, and cIMT

	Unstandardized coefficient	Standardized coefficient (β)	*p* value
CSBP	1.15± 0.43	0.12	.008*
CDBP	0.28 ± 0.31	0.04	.372
CPP	0.87 ± 0.35	0.14	.013*
CMAP	0.57 ± 0.31	0.08	.072
Ln AP	‐0.03 ± 0.03	‐0.05	.400
AIx75	‐1.12 ± 0.53	‐0.13	.036**
baPWV	‐21.48± 7.61	‐0.11	.005*
cIMT	‐0.07± 0.05	‐0.09	.121

*Abbreviations*: CSBP, central systolic blood pressure; CDBP, central diastolic pressure; CPP, central pulse pressure; CMAP, central mean arterial pressure; AP, augmentation pressure; AIx75, augmentation index standardized for a heart rate of 75 beats per minute. baPWV, brachial ankle pulse wave velocity analysis; cIMT, carotid intima media thickness; FBG, fasting blood glucose; TC, total cholesterol; UA, uric acid; peripheral MAP, peripheral mean atrial pressure; WC, waist circumference.

*Statistically significant after adjusting for sex, age, smoking status, alcohol consumption, TC, FBG, WC, UA, HR, peripheral MAP, antihypertensive therapy and lipid‐lowering therapy.

**Statistically significant after adjusting for sex, age, smoking status, alcohol consumption, TC, FBG, WC, height, UA, antihypertensive therapy, lipid‐lowering therapy and peripheral MAP.

### 3.3 Associations of urinary sodium excretion with baPWV and CIMT

Partial correlation analysis showed that estimated 24 h urinary sodium excretion was negatively correlated with baPWV (*p* = .005) but it was not correlated with CIMT (*p* = .168) (Table 2).

Multiple linear regression analysis showed that 24 h urinary sodium excretion was independently and negatively associated with baPWV (β = ‐21.48, *p* = .005) (Table 3).

**FIGURE 1:**
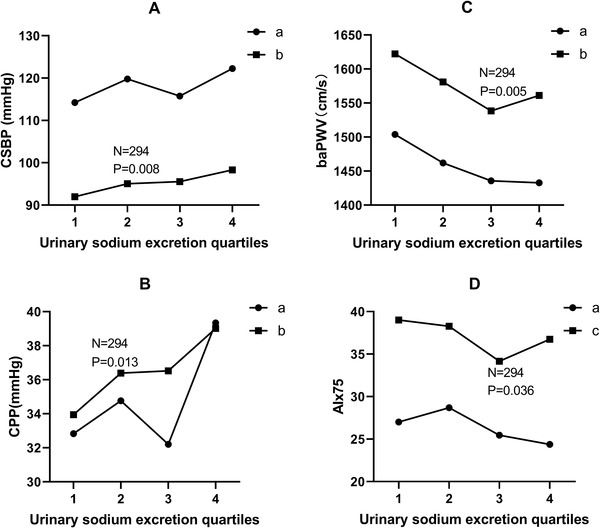
The associations between estimated 24 h urinary sodium excretion with CSBP (A), CPP (B), baPWV (C), and AIx_75_ (D). a: unadjusted. b: adjusted for sex, age, smoking status, alcohol consumption, TC, FBG, WC, UA, HR, peripheral MAP, antihypertensive therapy and lipid‐lowering therapy. c: adjusted for sex, age, smoking status, alcohol consumption, TC, FBG, WC, height, UA, peripheral MAP, antihypertensive therapy and lipid‐lowering therapy. CSBP: central systolic blood pressure; CPP: central pulse pressure; AIx_75_: augmentation index standardized for a heart rate of 75 beats per minute; baPWV: brachial ankle pulse wave velocity analysis. MAP: mean arterial pressure; FBG: fasting blood glucose; TC: total cholesterol; UA: uric acid; WC: waist circumference; HR: heart rate

After controlling for confounders, line graph of means across 24 h urinary sodium excretion quartiles showed that there was a positive linear association between 24 h urinary sodium excretion with CSBP and CPP. Associations of 24 h urinary sodium excretion with baPWV and AIx_75_ presented “J” shape as shown in Figure 1.

## 4 DISCUSSION

Findings of the current study established that estimated 24 h urinary sodium excretion of native general Tibetans living in Luhuo County was 5.26±1.61 g and estimated 24 h salt intake was 13.36±4.09 g. In addition, the current study showed that estimated 24 h urinary sodium excretion was independently and positively associated with CSBP and CPP. The current study also established that association of 24 h urinary sodium excretion with baPWV and AIx_75_ presented “J” shape.

Central blood pressure was reported to be more closely related to coronary perfusion and CV events compared with peripheral blood pressure. So far, few studies have explored association between 24 h salt intake with central blood pressure. Han and coworkers^12^ found that 24 h urinary sodium excretion was positively correlated with CSBP, CDBP and CPP in 431 hypertensive patients (24 h urinary sodium 3.83±1.61 g) from Beijing and Shandong. Furthermore, Sungha Park and coworkers^13^ showed that 24 h urinary sodium was independently and positively associated with CPP and AP in 515 hypertensive patients (24 h urinary sodium 3.45±0.92 g) from Korea. Michelle Redelinghuys and his colleagues^14^ recruited randomly 635 patients and collected 24 h urinary sodium, and they found that urinary Na^+^/K^+^ ratio was independently related to CSBP and CPP, but not CDBP, in general population. Similarly but not identically, the current study indicated that 24 h urinary sodium was positively associated with CSBP and CPP in general Tibetans, suggesting that salt restriction intervention might be beneficial for control of central blood pressure. In the current study, 1 g increase in urinary sodium excretion was associated with 1.7 mm Hg increase in CSBP after controlling for age, sex, smoking, alcohol consumption, WC, HR, UA, FBG, TC and 1.2 mm Hg after additionally controlling for peripheral MAP.

Current medical guidelines lack recommendations for lower limits of salt intake. Furthermore, relevance of “the lower, the better” clarion for salt intake is still debatable. O'Donnell and coworkers^15^ reanalyzed two cohorts (n = 28880) included in ONTARGET and TRANSCEND trials and reported 24 h urinary sodium of 4.77±1.61 g/day estimated by morning spot urine (Kawasaki formula). Their findings indicated that association between estimated 24 h urinary sodium with CV events was J‐shaped. Sodium excretions of > 7 g/day and < 3 g/day were associated with increased risk of CV events after a median follow‐up of 56 months compared with sodium excretion of 3–5.99 g/day. However, both trials included patients at high risk of CV events. Therefore, biases could not be eliminated because many of the patients had CV diseases and were likely to reduce their salt intake informed by doctors or media. In 2014, O'Donnell and coworkers^16^ enrolled 101,945 patients in PURE study from 17 low‐, middle‐, and high‐income countries, most of whom had no history of CV diseases. Estimated 24h urinary sodium excretion from morning spot urine of these patients was 4.93 g/day (Kawasaki formula). After median follow‐up of 3.7 years, the observational study also indicated that individuals with urinary sodium excretion of 3–6 g/day had the lowest incidences of mortality and CV events. The study also showed that association between high urinary sodium excretion and CV events was only significant in patients with hypertension and such relevance weakened when blood pressure was adjusted, indicating that high salt intake contributed to CV diseases by elevating blood pressure. Nevertheless, in low sodium excretion group, the association was evident in patients with or without hypertension and remained significant when controlling for blood pressure, implying that low salt intake led to high risk of CV diseases independent of blood pressure. To our knowledge, arterial stiffness is closely related to CV diseases. Therefore, exploratory studies on relationship between urinary sodium excretion with arterial stiffness could provide clues for association of salt intake with CV events.

Association between urinary sodium excretion with arterial stiffness has rarely been reported and most previous studies focused on patients with hypertension or obesity. Few studies has been undertaken in general population or non‐hypertensive people and yielded quite different results. Seung Ku Lee and coworkers^17^ established that urinary sodium excretion was independently and negatively correlated with baPWV and CIMT in a Korean genetic epidemiology study, including 1586 non‐hypertensive people with average estimated urinary sodium excretion of 3.6 g. They speculated that J‐shaped association was not observed due to low urinary sodium excretion. Another study^18^ undertaken in 630 Flemish general people evaluated daily sodium intake (3.9 g/day) by collecting 24 h urine samples and reported that urinary sodium concentration, rather than 24 h urinary sodium, was negatively related to AI. Luis García‐Ortiz and his colleagues^19^ reported J‐shaped associations of sodium intake (24 h sodium 3.18±1.25 g) with PWV and IMT in 351 Spanish general population after controlling for age, sex, cigarette smoking, alcohol consumption, medication and 24 h SBP. Their study excluded patients with history of CV diseases but assessed sodium intake using food frequency questionnaire which may have deviated significantly from actual status of salt intake. The current study excluded patients with history of CV diseases and natives had an extremely low awareness rate of benefits of salt restriction, indicating that they were less likely to deliberately restrict salt intake. Twenty‐four hour urinary sodium excretion was estimated from a second spot urine in the morning by Kawasaki formula. After adjusting for sex, age, smoking status, alcohol consumption, WC, height, peripheral MAP, TC, HR, FBG, UA, antihypertensive therapy and lipid‐lowering therapy, associations of estimated 24h urinary sodium excretion with baPWV and AIx_75_ presented “J” shape, which was similar to relationship of urinary sodium excretion with CV events established by O'Donnell and coworkers Lowest point of J curve was mildly higher compared with previous reported “safe” zone of sodium intake in a study by O'Donnell (3–6 g/day), we speculated that the observed deviation might be attributed to three reasons: First, spot urine samples were only collected once, which did not exclude day‐to‐day variation of urinary sodium excretion. Second, applicability of Kawasaki formula for estimating actual 24 h sodium intake in the studied population is unclear. Third, long‐established high salt diet due to alpine and cold climate as well as special Tibetan genetic profiles might have rendered locals more tolerable to detrimental effects of high salt intake compared with people living in plains. This implies that safe zone of sodium intake might have been mildly higher.

In the current study, linear relationship of urinary sodium with CSBP and CPP, as well as J‐shaped associations of urinary sodium with baPWV and AIx75 were not affected by hypoxic environment in Luhuo County, because similar findings have been reported by previous studies conducted in low altitude areas. Permanent settlement at high altitude area may have invoked different physiological adaptations among residents including vascular function. Previous studies showed that healthy Andean highlanders and Sherpa presented similar or only slightly lower vascular functions compared with lowlanders at sea level.^20^, ^21^ Furthermore, previous studies speculated that hypoxia‐induced increases of hemoglobin levels, blood viscosity and shear stress also induced production of vasodilator nitric oxide (NO).^22^ This balance may be an essential adaptation of preserved vascular function. Therefore, these complicated adaptations reduce effects of hypoxic environment in the current study on associations of urinary sodium excretion with central blood pressure and vascular changes.

Mechanisms of arteriosclerosis caused by high salt intake have been well‐established in previous studies. High salt activates renin‐angiotensin‐aldosterone (RAAS) system and sympathetic system and inhibits synthesis of nitric oxide produced by endothelial cells, which are closely associated with arteriosclerosis.^1^, ^23^ However, mechanisms underlying association between low salt intake and arteriosclerosis are unclear. A Cochrane systemic review,^24^ which included 167 studies, indicated that salt restriction led to 3.5% reduction of blood pressure in hypertensive patients and only 1% reduction of blood pressure in general population. However, salt restriction also contributed to significant increase in plasma renin, aldosterone, norepinephrine and epinephrine as well as 2.5% increase in cholesterol and 7% increase in TG, all of which are closely related to arteriosclerosis. Another previous study by Sajadieh A and coworkers^25^ indicated that hyponatremia was positively correlated with mortality and CV events in 670 patients with no history of CV diseases, stroke and cancer after a median follow‐up of 6.3 years. In the current study, LDL‐C and TC levels were lowest in third quartile of urinary sodium excretion, TG levels were the lowest in second quartile group and HDL‐C levels were the highest in third quartile group. However, the trend was not statistically significant, which needs to be further clarified in large samples. The current study postulated that high salt intake elevated blood pressure and contributed to arteriosclerosis, which synergistically increased risk of CV events. However, benefits of low‐salt induced reduction of blood pressure were counteracted by detrimental effects of low salt‐induced arteriosclerosis, which also increased risk of CV events. Nevertheless, CV events are consequences of co‐action of multiple factors and arteriosclerosis is among potential mechanisms. Associations of salt intake with vascular changes and risk of CV events should be explored further by large randomized controlled studies.

The current study had some limitations. Firstly, although determination of 24 h urinary sodium excretion is the gold standard for assessing salt intake, it is difficult to undertake accurate collection and inconvenient to operate. The current study, therefore, adopted Kawasaki formula to estimate urinary sodium excretion by spot urine, which might have deviated from actual status of salt intake to some extent. However, the current study considered Kawasaki formula applicable in population study. Secondly, hormones, including renin, angiotensin, aldosterone, and catecholamine, were not determined for practical reasons, which should be improved in future studies to explore potential mechanisms. Thirdly, inherent nature of cross‐sectional study confined explanation of causal relationships between urinary sodium excretion and vascular changes. Large randomized controlled studies are needed to overcome these limitations.

Based on findings of the current study, dietary salt restriction is essential for control of central blood pressure considering current status of high salt intake in this high‐altitude area. J‐shaped association between urinary sodium with vascular changes should be confirmed in future studies by enlarging sample sizes from more sites in high altitude Tibetans area and collecting 24 h urine samples. A recent study revealed that daily pattern of urinary sodium excretion, aside from the amount of sodium excretion, was associated with central blood pressure and arterial stiffness.^26^ This implies that intraindividual pattern of urinary sodium excretion should also be explored in future studies to improve individualized risk stratification and management of hypertension and other CV diseases.

## CONFLICT OF INTERESTS

On behalf of all authors, the corresponding author states that there is no conflict of interest.

## AUTHOR CONTRIBUTIONS

Xiaoping Chen, Zhipeng Zhang, Hang Liao and Xin Zhang are responsible for designing the study, selecting the research location, communicating execution details with local CDC staffs. Zhipeng Zhang, Hang Liao, Xin Zhang, Qingtao Meng, Rufeng Shi, Jiayue Feng, Xinran Li, Qiling Gou, Runyu Ye and Xianjin Hu participant in performing the examinations for study patients. Zhipeng Zhang are responsible for writing and editing the manuscript. Hang Liao are responsible for editing the manuscript.

## Supporting information

Supporting material
