# Supplementary material for: Associations of urinary sodium excretion with central hemodynamics and changes in vascular structure and function at high altitude
Source: J Clin Hypertens (Greenwich). 2021 Sep 3;23(10):1907–14. doi: 10.1111/jch.14356 (PMC8678796; doi:10.1111/jch.14356)
Supplement: Supplementary file 1 — Supporting material [file JCH-23-1907-s001.docx]

**Related formula**

Central hemodynamic parameters:

CPP = CSBP – CDBP. AP = the amplitude difference between the second peak and the first peak of the aortic wave form in systolic period. AI = AP / CPP.

Kawasaki formula:

Estimated 24h urinary Na (g/d) = 23×0.001×16.3× (Na (mmol/l) / Cr (mg/dl×10) × predicted 24h urinary Cr (mg/d)) ^0.5^;

Predicted 24h urinary Cr (mg/d) (female) =-4.72×age (year) + 8.58 × weight (kg) + 5.09 × height (cm)-74.5；

Predicted 24h urinary Cr (mg/d) (male) =-12.63 × age (year) + 15.12 × weight (kg) +7.39 × height (cm)-79.9.
